# An Artificial Intelligence Approach for Expurgating Edible and Non-Edible Items

**DOI:** 10.3389/fpubh.2021.825468

**Published:** 2022-01-27

**Authors:** Dilip Kumar, Urvashi Bansal, Roobaea S. Alroobaea, Abdullah M. Baqasah, Mustapha Hedabou

**Affiliations:** ^1^Department of Computer Science and Engineering, Dr. B. R. Ambedkar National Institute of Technology, Jalandhar, India; ^2^Department Computer Science, College of Computers and Information Technology, Taif University, Taif, Saudi Arabia; ^3^Department of Information Technology, College of Computers and Information Technology, Taif University, Taif, Saudi Arabia; ^4^School of Computer Science, Mohammed VI Polytechnic University, Ben Guerir, Morocco

**Keywords:** food sanitization, smart sanitization, UVC, germicidal, food safety, machine learning for health, smart system

## Abstract

In the pandemic of COVID-19, it is crucial to consider the hygiene of the edible and nonedible things as it could be dangerous for our health to consume infected things. Furthermore, everything cannot be boiled before eating as it can destroy fruits and essential minerals and proteins. So, there is a dire need for a smart device that could sanitize edible items. The Germicidal Ultraviolet C (UVC) has proved the capabilities of destroying viruses and pathogens found on the surface of any objects. Although, a few minutes exposure to the UVC can destroy or inactivate the viruses and the pathogens, few doses of UVC light may damage the proteins of edible items and can affect the fruits and vegetables. To this end, we have proposed a novel design of a device that is employed with Artificial Intelligence along with UVC to auto detect the edible items and act accordingly. This causes limited UVC doses to be applied on different items as detected by proposed model according to their permissible limit. Additionally, the device is employed with a smart architecture which leads to consistent distribution of UVC light on the complete surface of the edible items. This results in saving the health as well as nutrition of edible items.

## 1. Introduction

Health is the most critical factor in our life and majorly depends upon what we eat and what is the quality of our daily intake which includes fruits and vegetables having essential minerals, vitamins, and proteins (1). The raw vegetables and fruits may have viruses or pathogens on their surfaces even if some of them are removed by a wash. Due to the COVID-19 pandemic, this becomes essential that the things we are intaking are pathogens free and are virus-free whether it is edible items or our daily usable items. There are many products available in the market for sanitizing edible and non-edible items e.g., UVC rays (2–4). It is already proven that germicidal UVC has properties that deactivate the pathogens by exposing the surface of fruits and vegetables for a few minutes (5–8).

In recent times, it is seen that researchers are widely using UVC to perform complex operations such as scientific applications for deactivating viruses; water purification to remove viruses and bacteria from the water; air purification which deactivates the viruses and germs present in the air of the atmosphere (9); sanitizing a room. Additionally, UVC has many applications for deactivating pathogens over different surfaces and mediums. It is also being used with fluorescent which makes the viruses glow when it is seen by microscopes in presence of the UVC rays. UVC is cheap and easily available in the market which further increases its usage.

In the existing solution, there is a limitation that it damages approximately 30% of the edible items especially fruits and vegetables as its being used by industries for decades for removing the pathogens present on the surface of these items (10–12). So, the existing solution does not have an automated system that can identify the object and make optimal configuration accordingly and an existing solution does not have any filter which prevents the damage done by the exposure of the direct UVC to this item.

We have designed an Artificial Intelligence employed device to provide an automated self-learning process in which the object is identified by the device itself by using machine learning and make optimal configurations as per the class of the object classified by the object detection model. So, AI enables automation (13) with the optimal setting configuration and makes the device smarter than the existing solutions.

## 2. Our Contribution

In the view of limitations of existing solutions, we have incorporated certain changes in the proposed device so that edible items do not loose its nutrient values.

- We have used an AI-Enabled UVC sanitization device powered by raspberry pi 4, UVC light 254 nm with polarizing filters for the limited dose to the fruits and vegetables, and a camera sensor for object detection.
- We have used the rotating base to apply UVC light uniformly throughout the object.
- The vacuum pump is used to remove dust particles from inside the box which further maintains the uniform intensity of UVC.
- Inside the box, the mirror is presented on each side so total light reflection occurred when the rays from the UVC lamp strikes the mirrors at different angles.
- The aluminum sheet is also sandwiched between the outer shell and the mirror for maximum heat dissipation when the heavy dose is required by the objects.
- Bipolar fans are also present to cool down the aluminum sheets and can dissipate the heat properly out of the box.

We have used machine learning models for automated UVC sanitization. There are many different objects like vegetables and fruits which vary with proteins structure and have different molecules. It is necessary to detect the object placed inside the box so a camera sensor is used to capture the image of the object placed. This object image is passed to the machine learning model which detects the class of the object and then this class is passed to another classifier which classifies the rotation angle and the safe exposure time for that object based on the similarity.

## 3. Literature Review

UVC Sanitation is being used for many decades in the industry (14). It is not a new thing that is used for sanitizing the different edible and non-edible items. The industry is using germicidal UVC for raw fruits and vegetables to deactivate the pathogens present on the surface of vegetables and fruits (8). Germicidal UVC of 254 nm has the capability of deactivating pathogens as well as air-born virus-like h1n1 flu and also COVID-19 virus (2, 3, 6, 15). Recently, many devices have come into the market which claims that their devices can sanitize any object. There are many products available in the market like air purifiers using the germicidal UVC.

UVC Sanitization box is available in the market in different sizes which can sanitize the items using the germicidal UVC of 254 nm. There is also a UVC device available that can be used for sanitizing small electronic devices and non-electronics devices (5). These devices are good enough for sanitizing the electronic device and the non-edible items but they are not much effective for edible items like raw vegetables and fruits (3).

Some of the findings were provided in Table 1. As discussed in existing literature, there is a limitation that a few minutes of doses can lead to damage to edible items like fruits and vegetables. So, we proposed a novel idea to overcome this limitation.

**Table 1:** Existing findings.

**Author**	**Objectives**	**Finding**
Weber et al. (16)	To see how effective Ultraviolet technologies are at reducing microbial contamination on environmental surfaces in patient care environments.	UV sanitization is more likely to be unaffected in darker places.
Scotland (17)	Analyzed the scientific evidence supporting UV light disinfection systems efficiency.	The following factors influenced the effectiveness of UV light: organic load and pathogen; the strength and amount of UV light; the distance from the device; exposure time; surface to be cleaned is within direct line-of-sight or not.
Barbut et al. (18)	The eradication of bacterial spores using hydrogen peroxide sprays and sodium hypochlorite solution was studied in a prospective, randomised, prior to actually trial.	The eradication of bacterial sporesa utilising hydrogen peroxide sprays and sodium hypochlorite solution was studied in a prospective, randomised, before-and-after study. Hydrogen peroxide sprays were shown to be significantly more effective than sodium hypochlorite solution at killing Clostridium difficile spores. The later has the potential to be a viable tool for eradicating Clostridium difficile spores.
Holmdahl et al. (19)	On biological markers, the tests evaluated the effectiveness of hydrogen peroxide and sodium hypochlorite.	On G. Stearothermophilus bioindicators, hydrogen peroxide vapour generators were faster and more effective than sodium hypochlorite machines.
Fu et al. (20)	A comparison of the safety and effectiveness profile of H_2_ O_2_ sprays against aerosolized hydrogen peroxide on G. Stearothermophilus biological indicators with MRSA, C. Difficile, with Acinetobacterbaumannii discs	The water vapour system has demonstrated a higher level of safety, speed, and efficacy in bacterial inactivation.
Haas et al. (21)	By comparing the frequencies of hospital-acquired MDROs before and after UVD use, a retrospective analysis of the efficiency of UV light environmental decontamination as a complement to improved terminal cleaning of rooms formerly occupied with isolated patients was conducted.	Even though nearly a quarter of the disinfection chances were missed, there was a considerable drop in hospital-acquired MDRO rates during the UVD usage period compared to the previous period. UV technologies appeared to have some positive effects in this investigation.
Anderson (5)	On the surfaces of segregation units, the antibacterial capabilities of UVC light and chemical sanitisers were compared.	UVC proved ineffective in dark portions of the rooms, required further chemical decontamination.
Memarzadeh et al. (22)	The importance of UV light technology in air purification in a healthcare setting is discussed.	UV technologies cannot yet be applied to neutralise or eliminate germs as a stand-alone intervention, but they can be employed in conjunction with other traditional treatments for endpoint disinfection.

## 4. Methodology: System Architecture Overview

### 4.1. Architecture

**Figure 1:**
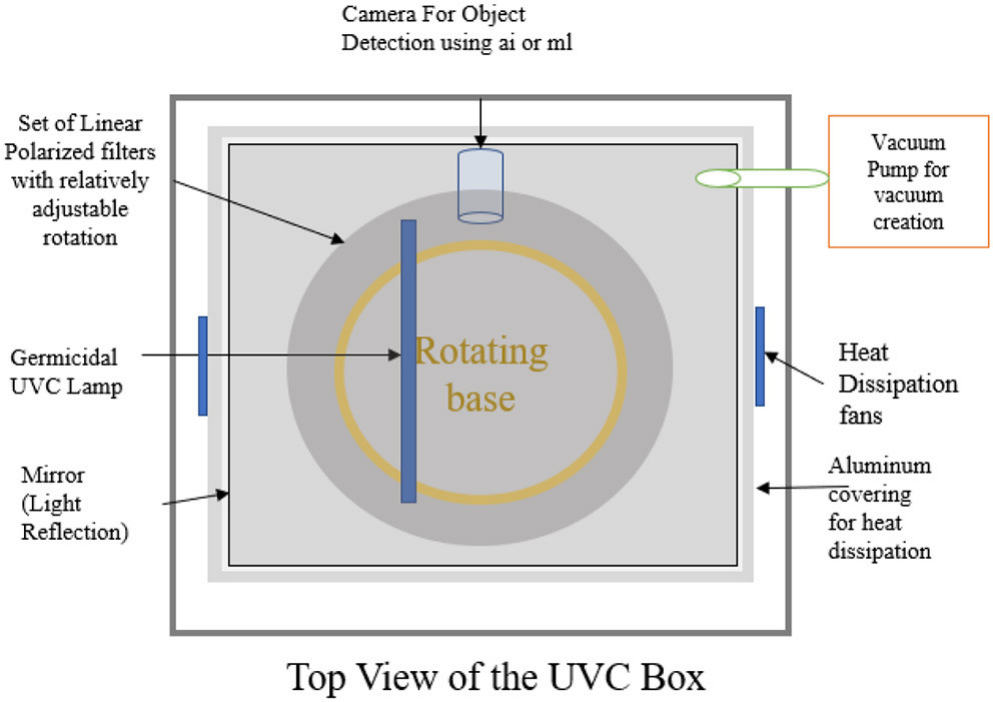
Top view of the UVC device.

**Figure 2:**
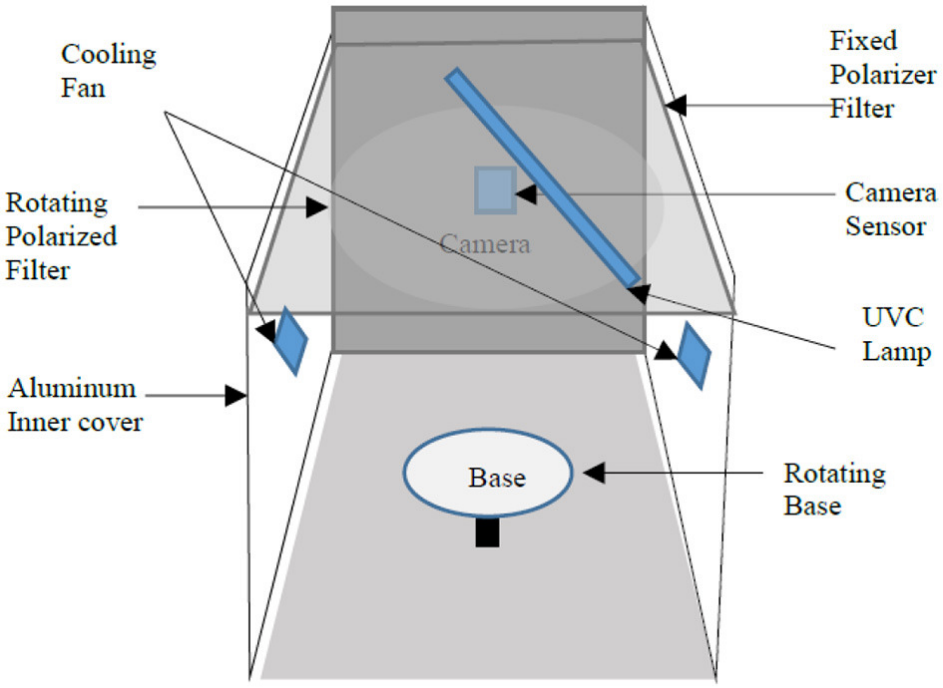
Front view of the UVC device.

The newly built proposed design of the AI-enabled UV sanitization box, as shown in Figures 1, 2, depicts the proposed architecture with the following units:

- **Processing unit:** Raspberry pie 4 would be used as a processing unit.
- **Object detection unit:** This is the unit where all the images captured by the image sensor are pre-processed and then the image of the object is passed to the AI trained model which detects the category of object and results in the category of the object. Here, we have created a machine learning model using the CNN and we trained the model with training images of the different fruits and vegetables.
- **Sensors:** We have used a camera module as an image sensor and temperature sensor along with a cooling module.
- **Alarm unit:** We have used speakers and voice enable system to alert the user.
- **Cooling unit:** The same purpose is solved with heat sink and cooling fans.
- **UVC rays filter unit:** UVC 254 nm is very harmful to the human skin as well as it can damage fruits and vegetables. So, to avoid that damage to the vegetables and fruits, the intensity of the light is controlled using the two polarized sheets (23– 26). One sheet is fixed and one sheet is made to rotate on both sides directly with the help of a stepper motor. The range of the rotation varies from 0 to 180_0_. Intensity can be controlled between 0 and 180_0_ from total light pass to total blockage of the light through the polarizing filter (27).
  - **Parallel axis:** When two sheets are placed parallelly, they allowed the total light to passed through it.
  - **In between axis:** If one sheet is fixed and rotation of the sheet is started in either direction, it will start blocking the light as the sheets are getting crossed to each other. If further rotated, it starts allowing the light through it.
  - **Crossed axis:** If two sheets are crossed then it blocks the light passing through it.
- **Rotating base unit:** A rotating base is used for the even distribution of the UVC rays to the object which is placed on the rotating base. It also prevents the direct penetration of the UVC. The base starts rotating with the start of the sanitization process.
- **Display unit:** The display unit displays all events that occurred at the device. It starts from the device start message and mode selected and then displaying the timer set for the UVC sanitization up to the end of the sanitization process.

### 4.2. Mechanism of Sanitization

Smart UVC Sterilization has a special mechanism of sanitization. It is operated from 230-volt AC. There are two shields: one is the outer shell and one is the inner shell. The opening door consists of an auto cut UVC power supply mechanism which is operated using a switch. It will auto cut the power source to UVC when we open the door and a visible lamp will glow to put the objects inside the UVC box. It has a rotating plate that carries the objects. We have a few pre-set items on the operational menu. We can use those pre-sets if we know the edible item otherwise the auto mode is selected.

**Figure 3:**
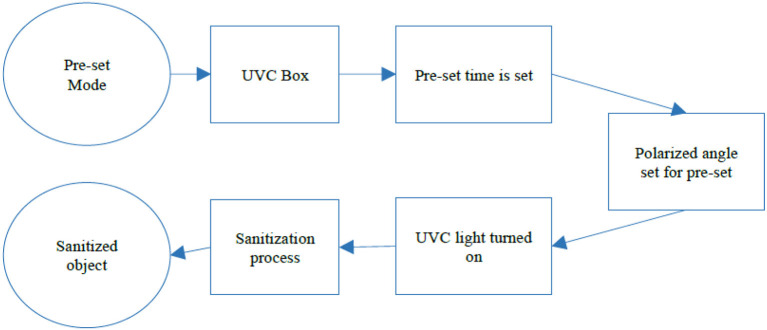
Pre-set mode operation of UVC sanitization.

**Figure 4:**
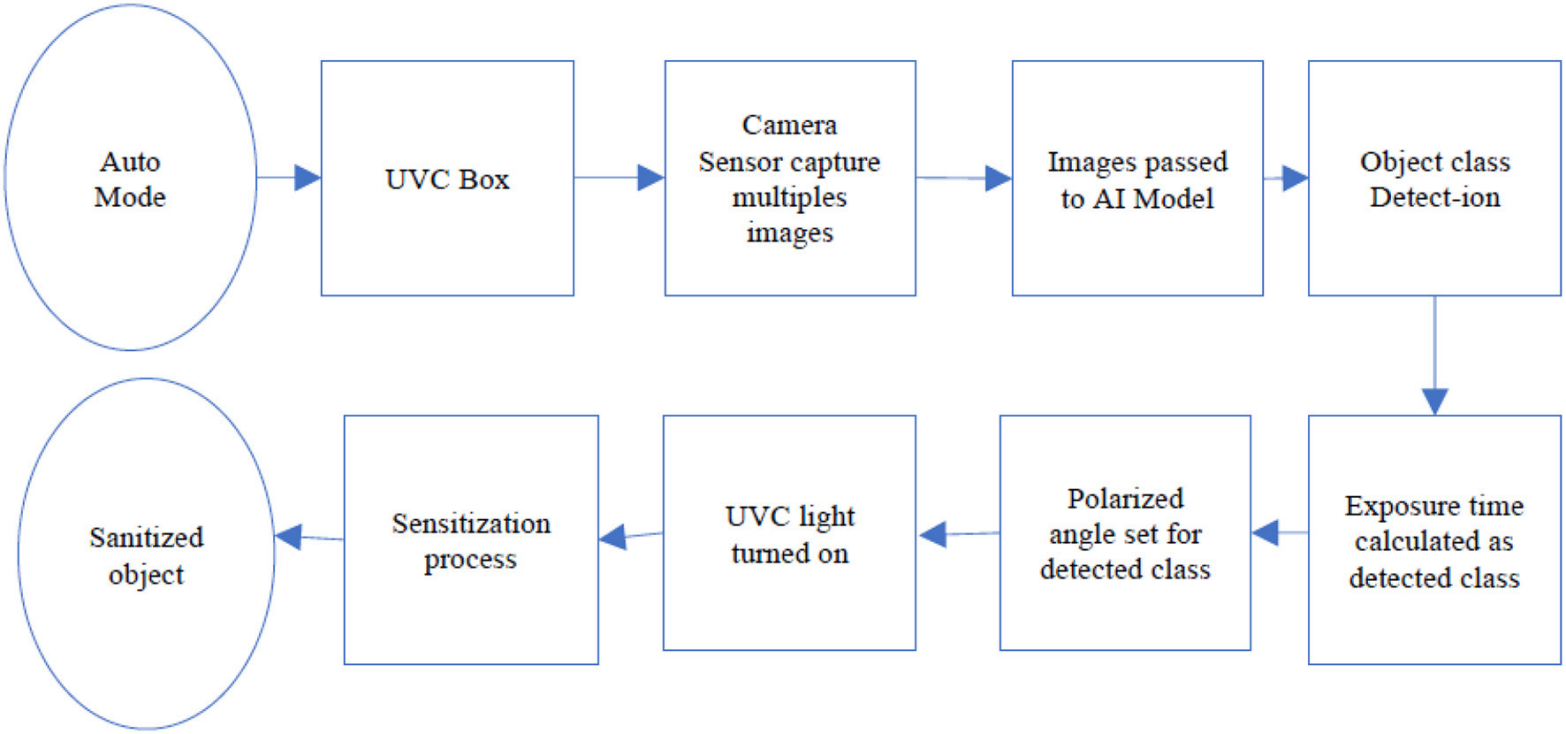
Auto mode operation of UVC sanitization.

When the pre-set mode is selected, the UVC Box is set to the predefined fixed mode where the UVC box works on the predefined values for the UVC exposure times and the UVC box runs for the predefined time and set its polarized angle as defined for that mode (ref Figure 3). In the Auto mode, the UVC box uses the AI model to detect the object inside the box. The process is shown in Figure 4.

## 5. Control Design

Control design process of the AI-enabled UVC device consists of different process flows and control structures used in building the device and process description which defines the different sequences of processes held in the device.

### 5.1. Main Process Building Block

**Figure 5:**
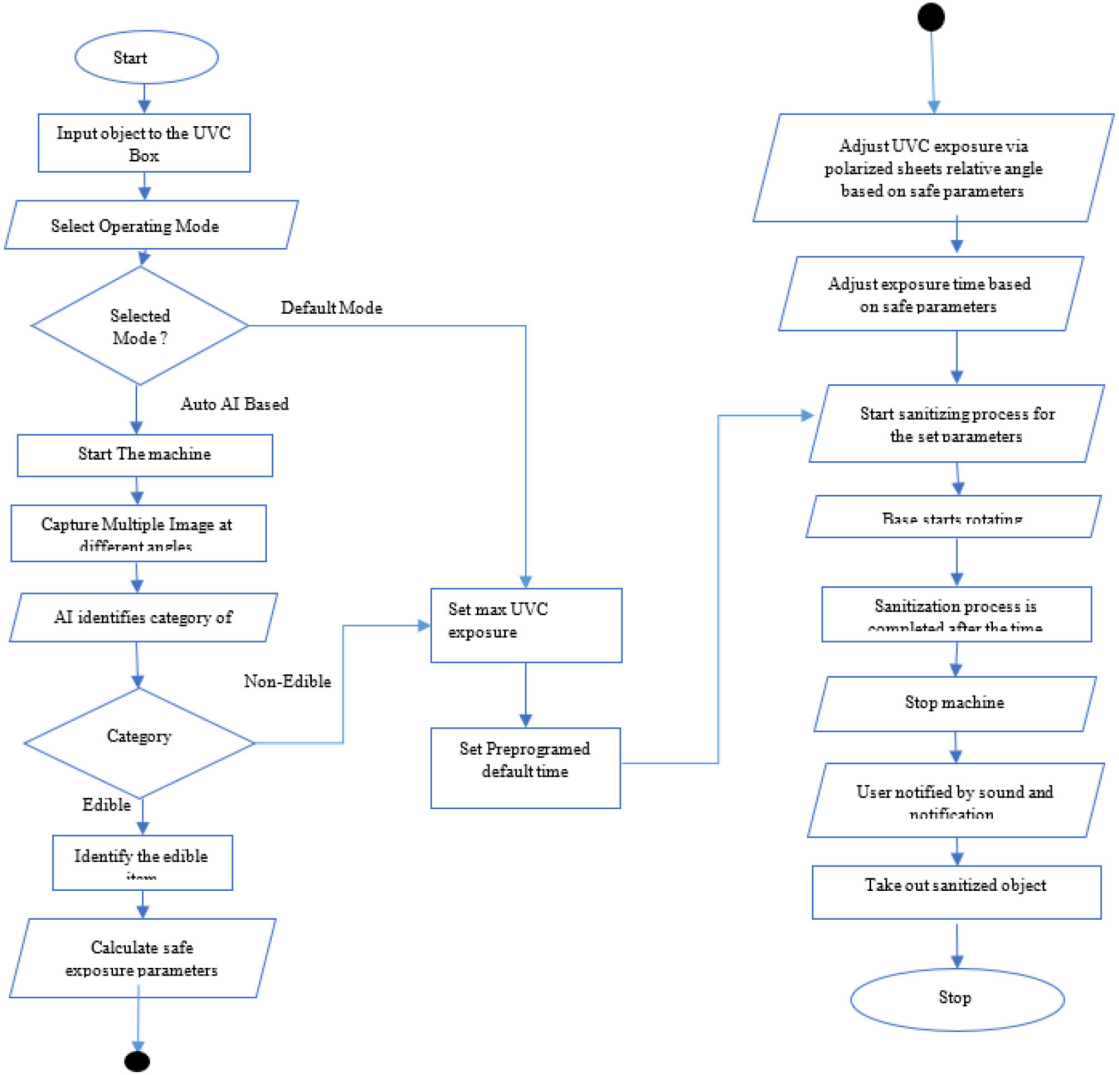
Main process flow diagram.

This section defines the process of the overall workflow of the AI-Enabled device (28). It defines the overall process of the device from the start to the end of the sanitization process. The main process block is defined in Figure 5. The process starts from inputting the edible or non-edible object inside the device and then needs to select the mode of operation of sanitization.

There are two modes: one is manual which can be used directly for the non-edible items; second mode is auto mode which once selected, it starts capturing the images of the object inserted inside the object. After successfully capturing the image, these images are passed to the object detection model which detects the class of the object and the required UVC exposure time is stored using the UVC dosage calculation formula given in equation 1. According to equation 1, we can easily determine that the total UVC dose time is calculated on the basis of the intensity of light dropped for the particular amount of time.


(1) $$\begin{array}{r} UVC\text{\_}Dose=UVC\text{\_}rays\text{\_}Intensity\left(\mu W/cm{}^{\text{2}}\right)\\ \;\times UVC\text{\_}Exposure\text{\_}Time\left(seconds\right). \end{array}$$

On the basis of Table 2, we have calculated the expected doses for the predicted item class. If the class of the object is detected to edible items, then the safe exposure parameters are set according to the detected object, and the rotation angle of the polarizing filter and time of UVC rays exposure is measured and stored using another classifier that classifies the edible item based on the few parameters. Finally, the sanitization process gets started. The procedure is provided in Algorithm 1.

**Table 2:** UV-C doses for SARS-CoV-2.

**Viral inactivation (%)**	**UV-C dose (mJ/cm^2^)**	**Exposure time (s)**
90	0.016	0.01
99	0.706	0.32
99.9	6.556	2.98
99.99	31.880	14.49
99.999	108.714	49.42

**Algorithm 1:** UVC_Process.

1 Intialise: object_class= “edible”, mode=0, uvc_time=0, filter_angle=0
2 Select the mode of operation and set mode equal to mode selected.
3 if mode = manual **then**
4 go to step 8
5 else **if** mode=auto **then**
6 go to the step 15
7 end **if**
8 Set the uvc_time to 300 s
9 Set the filter_angle equal to 0.
10 Rotate the polarizer sheet to angle equal to filter_angle.
11 Turn on UVC Lamp.
12 Start Rotating Base.
13 Run the Sanitization process for the set uvc_time.
14 go to step 21.
15 Capture an image of the input item.
16 Detect the class of the object using AI model and set object_class equal to detected object class.
17 Detect the filter angle and optimized UVC sanitization time using optimized setting_finder_model.
18 Set the uvc_time equal to detected optimized detected time
19 Set the filter_angle equal to the detected polaroid angle of rotation.
20 Rotate the filter upto the filter_angle.
21 Start the sanitization process for the set time equal to the uvc_time.
22 Stop UVC light on completion of the set time.
23 Rotate the polarizer back to its original position.
24 Stop Rotating base.

### 5.2. Control Process Flow of Camera Sensor

The Camera Sensor is used for capturing the images of the inserted object whenever the auto mode is selected. AI comes into the picture when auto mode is selected and there is a need to classify the class of the object inserted inside the device. Image captured by the camera sensor is saved to the raspberry pi and then it is transferred to the machine learning model which is already trained over the 131 classes of fruits and vegetables. The camera sensor helps to detect the class of the object inserted inside the device.

### 5.3. Object Detection Process Flow

The object detection process flow is the process that defines the classification of the input Image *via* a pre-trained model loaded to the raspberry pi. The object detection model is trained over 65,000 images with 131 classes. Image is passed to the model and the model predicts the class and returns the class of the object if it is predicted otherwise return nonedible.

### 5.4. Polarizer Filter Process Flow

The Polarizer filter process depicts the process of principle used for controlling the intensity of the UVC rays *via* changing the angle between two polarized sheets. One sheet is mounted with the stepper motor which rotates the motor in both directions. When two sheets are crossed to each other it is 180 degrees angle and zero for full light pass through the polarized sheet. The process starts with the configuration settings from the detected object class and starts rotating to the set angle for the detected class.

### 5.5. Cooling System Process Flow

The cooling system is an essential part of the device as it takes care of the heat generated inside the device and prevents the excessive heat generated by the UVC rays. To avoid damage from the heat generated by the UVC rays, the cooling system helps the aluminum covering to act as the heat dissipation heat sink (26, 29).

The process of the cooling system consists of a heat sensor module where the auto cut relay is programmed with a temperature setting. The cooling fans control the temperature of the device. The temperature is set to the desired value. The threshold value for the temperature is set to 30^0^C. Whenever the temperature reaches above 30_0_C, it turns on all fans connected to the temperature module. If the temperature goes below 30^0^C, it stops all the fans. This process continues whenever there is a temperature rise and down.

## 6. Circuit Design

The Circuit design includes the interfacing of the different hardwares. This section depicts the interfacing of the different hardwares used in this device.

### 6.1. 2004a LCD Display Interfacing

LCD 2004 is a parallel LCD display which is simple and a cost effective solution for adding display characters on a 20 ×4 white and high contrast white text upon a blue background/backlight. The display has 20 characters and 4 lines. It requires 6 data pins to be interfaced with the raspberry pi GPIO pins and other 6 pins with the ground and 5 volts.

**Figure 6:**
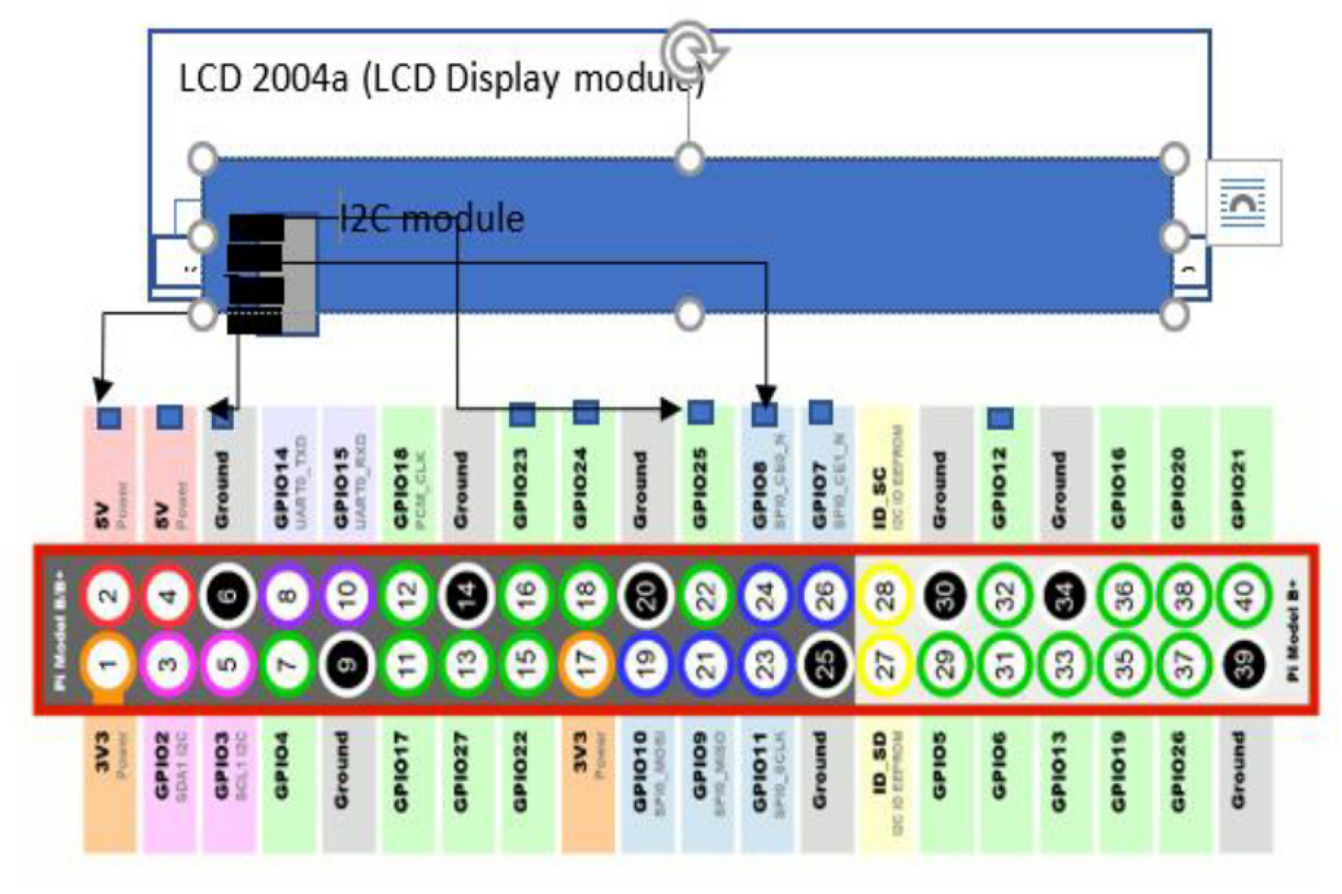
Interfacing of 2004a display LCD with Raspberry Pi.

The 2004a LCD is interfaced using the i2c adapter which reduces the connection wires from 16 to only a two-wire connection to the I2C adapter. I2C adapter supports many connections to be handled at a time as defined in Figure 6. So, it is used here to reduce the complexity of the raspberry pi. It reduces the number of GPIO pins used by the LCD.

### 6.2. NEMA 17 Stepper Motor Interfacing

**Figure 7:**
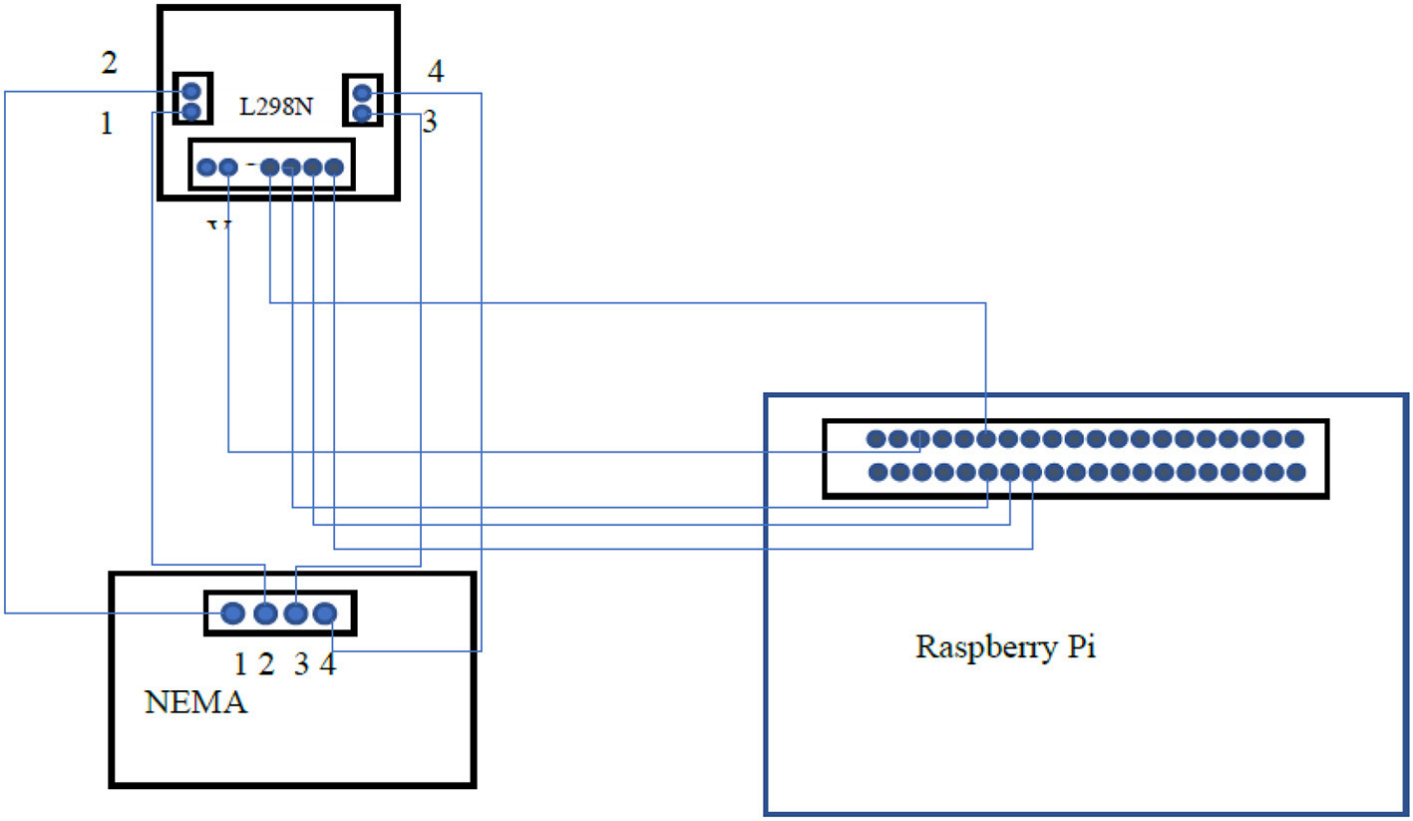
Interfacing of NEMA 17 stepper motor with Raspberry Pi.

The Nema17 stepper motor is used for rotating the object holding base. It has high torque which can rotate weight up to 4 kgs. The L298N stepper driver is interfaced with the Nema17 stepper motor and with a raspberry pi to drive the NEMA as shown in Figure 7. Stepper motor drivers need to be powered with 12 volts to drive the NEMA motor.

### 6.3. RJ2003 Stepper Motor Interfacing

**Figure 8:**
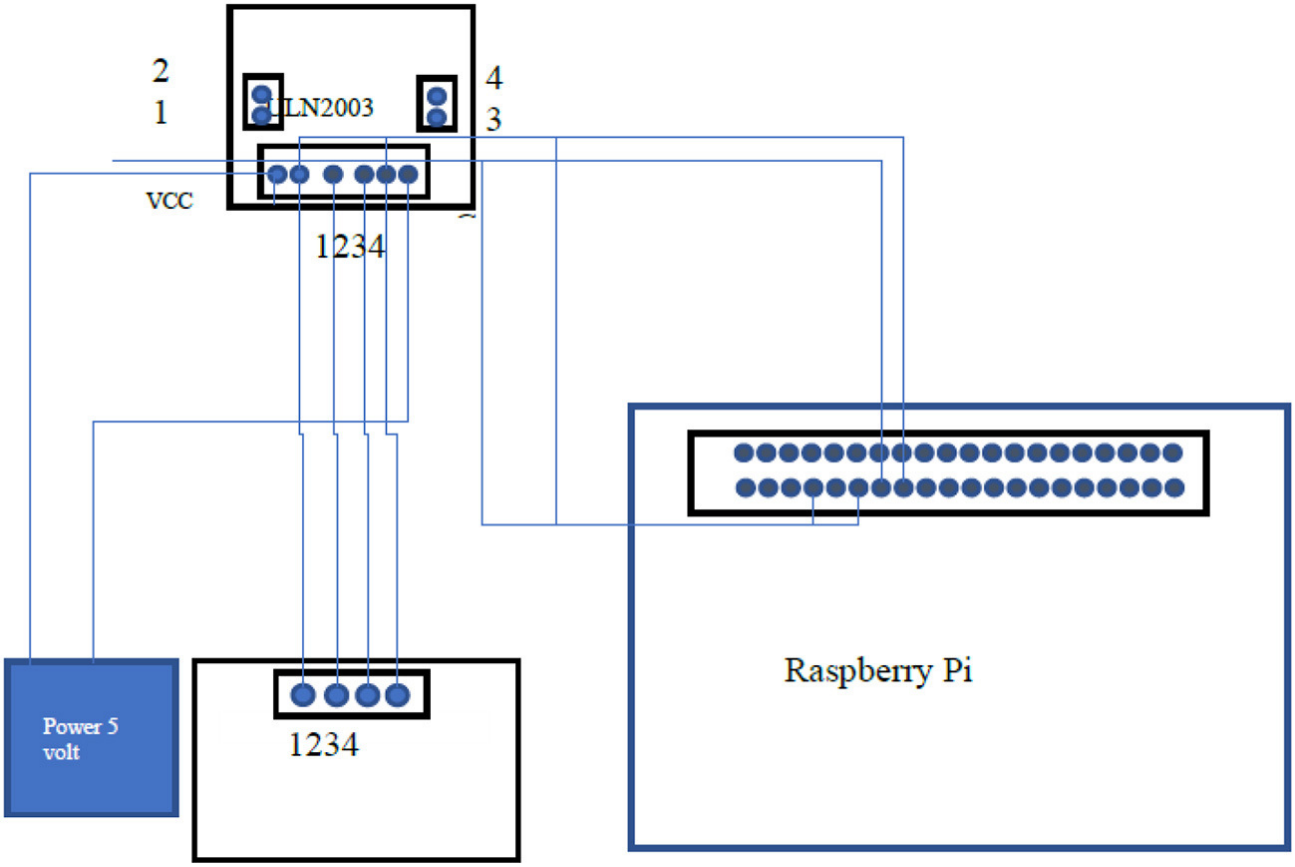
Interfacing of RJ2003 stepper motor with Raspberry Pi.

It is a stepper motor that consists of 4 coils which allow it to take very precise steps and very accurately it can rotate up to specific angles rotations. It is used for the rotation of the polarizer filter. It makes the filter from a 0-degree angle to 180-degree angle, which means from a total block of light to the total pass of the light through the polarizer filter. It is interfaced with the raspberry pi. In Figure 8, a stepper driver is used to drive the stepper motor that is ULN2003 which is interfaced with both the raspberry pi and stepper motor 28BY-J-48y.

## 7. Proposed Technique

### 7.1. Proposed Object Detection Model

We have used the deep learning method to train the model against the fruits and vegetable dataset of images. We obtained the dataset which is freely available named Fruits-360 which can be downloaded from GitHub or available at Kaggle. It includes about 130,000 images of different categories. This dataset includes high-definition images which are essential to obtain a good classifier (30–33).

**Figure 9:**
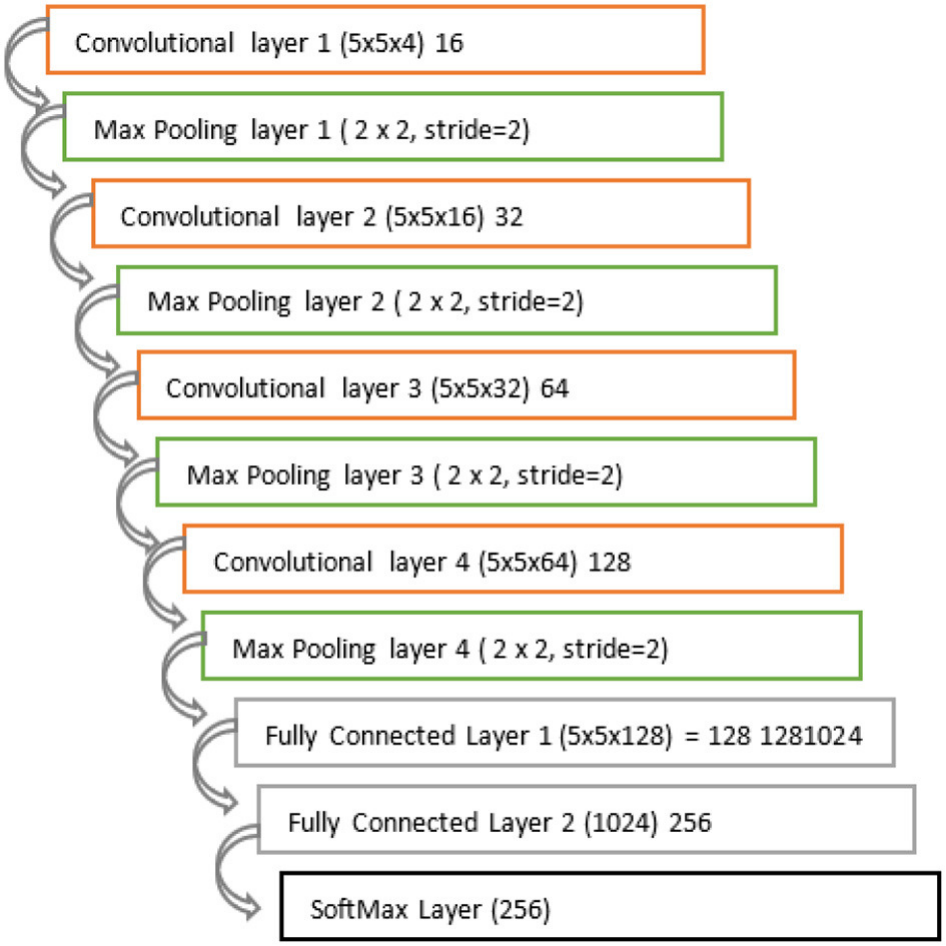
CNN model layers.

The structure of the model we used is defined in Figure 9. There are a total of 11 layers used in our model. There are four convolutional layers, 4max pooling layers and 2 fully connected layers, and the output layer is the SoftMax layer. The input layer is a convolutional layer where the filter of 16, 5 x 5 x 4 filter is applied and the max-pooling of 2 x 2 is used with stride=2. Convolutional layer 4 is with parameter (5 x 5 x 64) with dimension 128. The model is trained with the 131 classes of fruits and vegetables and it can be capable of detecting the multiclass of the image also.

### 7.2. Optimal Configuration Prediction Model

This is the model proposed to predict the optimal configuration for the detected object. This model makes a prediction based on the data collected through the experimentations. This collected dataset includes the entity name, its features and configuration such as time of UVC exposure and the angle of rotation of the polarizer.

The model used is a decision tree classifier and it works fine as per the requirement. We can classify the UVC exposure time and the angle of rotation of the polarizer and be able to detect the required configuration of the UVC sanitization process. This classifier gives an accuracy score of the 96.33%.

### 7.3. Smart Device Control

The device is controllable through a mobile app (android). There are two interface screens of the app. The use of the mobile app is to control the device using the Bluetooth communication channel and can be operatable using the mobile app interfaces (34–40). The wireless channel enables the message transfer from mobile to operate the device using the host program running on the raspberry pi and client program on the android app.

### 7.4. Control Screen

The device is configured for the following operations:

#### 7.4.1. Mode Selection

There are two select modes

- Auto mode
- Default mode

**Figure 10:**
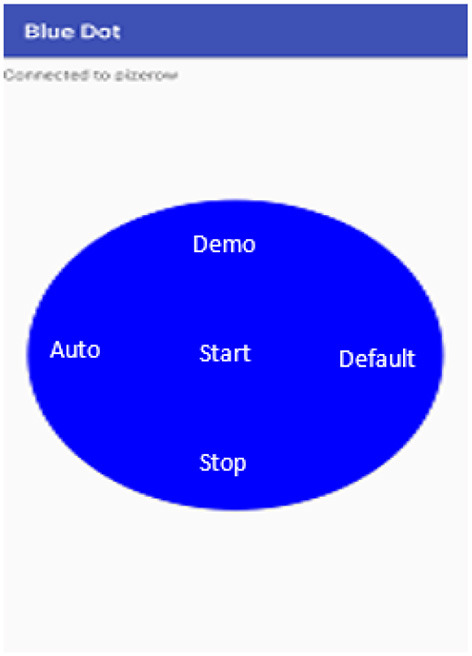
Mobile app main screen with controls.

The two modes can be selected from app as shown in the app screen in Figure 10. One of the mode is the auto mode where the AI comes into the picture and takes care of the rest of the process of the sanitization. The other mode is the default mode operating normally for non-edible items. Its configuration is fixed and loaded on the selection.

## 8. Results and Discussion

The code is implemented two times, one experiment is done with 20 items with 20 iterations, another one is done with the 25 items with 20 iterations. The goal is to create an optimal configuration for the discovered items.

### 8.1. Data Set

In this research realm, the two data sets taken are listed below:

- Fruits 360: This dataset includes the 131 classes of images of both fruits and vegetables (41).
- Configuration mapping: This dataset is made from the experiments performed by us for optimal configuration for the different objects.

### 8.2. System Requirements

The code of the proposed object detection and training model is written in Python and TensorFlow libraries. The code has been implemented to detect the object class and make optimal configuration using the classification model on experiments results to assess the performance of the UVC process algorithm. The following specifications are used to implement the algorithm:

- Processor - Intel(R) i7-8750H CPU @ 2.40GHz 2.40 GHz
- GPU - NVIDIA-GeForce GTX-1050
- RAM - 8 GB DDR4
- Hard disk drive - 256 SSD
- Operating System - Windows 10 Pro x64-bit

## 9. Results

The machine learning model used is CNN for object detection and we have used the fruits360 dataset (41) and 50 epochs for training the model which gives an accuracy of 96.2% without overfitting and underfitting as we have provided enough training data for training the model. We have trained the model on an i7 intel processor with Nvidia graphics as it is not possible to train a model over the raspberry pi. Due to limited hardware configuration, we trained it over the system and used the trained model in raspberry pi. So, the trained model gives a good result on the raspberry pi as it is trained over 131 classes of the fruits and vegetables of 65,000 images. We have run the device for the different configurations and the different filter parameters. We have run the machine for the following angle of filters as follows:

- 0^0^ angle filter rotation: Here, the light passes through the filters as the normal light passes as it permits the whole rays to pass through the filters.
- 45^0^ angle filter rotation: This angle of rotation allows lights to pass through the filter but with limitations as it does not allow the whole UVC light to get pass through it. It allows 75% of UVC light through.
- 90^0^ angle filter rotation: At this angle of rotation of the filter, 50% of rays are blocked and 50% of rays get passed through the filters.
- 135^0^ angle filter rotation: At this angle of rotation of the filter, 70% of rays are blocked and 25% of rays get passed through the filters.
- 180^0^ angle filter rotation: At this angle of rotation of the filter, 100% rays are blocked and 0% rays get passed through the filters.

So, we have used the 45-degree, 90-degree, and 135-degree rotations for our experiments and get the following results for a few of the fruits as shown in Table 3. We have calculated the UVC dose time using equation 1. As our UVC lamp has a UVC intensity of 300 μ*W*/*cm*^2^ and the length we have to use is 15 cm. As represented in Table 3, the edible items (especially raw fruits and vegetables) can be sanitized without losing their quality and without any damage to the cell body of the edible item using the proposed system architecture.

**Table 3:** Experimental results.

**Item**	**45^0^ Angle time(s)**	**Remark**	**90^0^ Angle time(s)**	**Remark**	**135^0^ Angle time(s)**	**Remark**
Apple	120	Ok	180	Ok	300	Ok
Banana	120	Ok	180	Ok	300	Ok
Grapes	120	Ok	180	damage	300	damage
Papaya	110	Ok	175	Ok	280	Ok
Kiwi	123	Ok	185	Ok	290	Ok

### 9.1. Performance Plot

**Figure 11:**
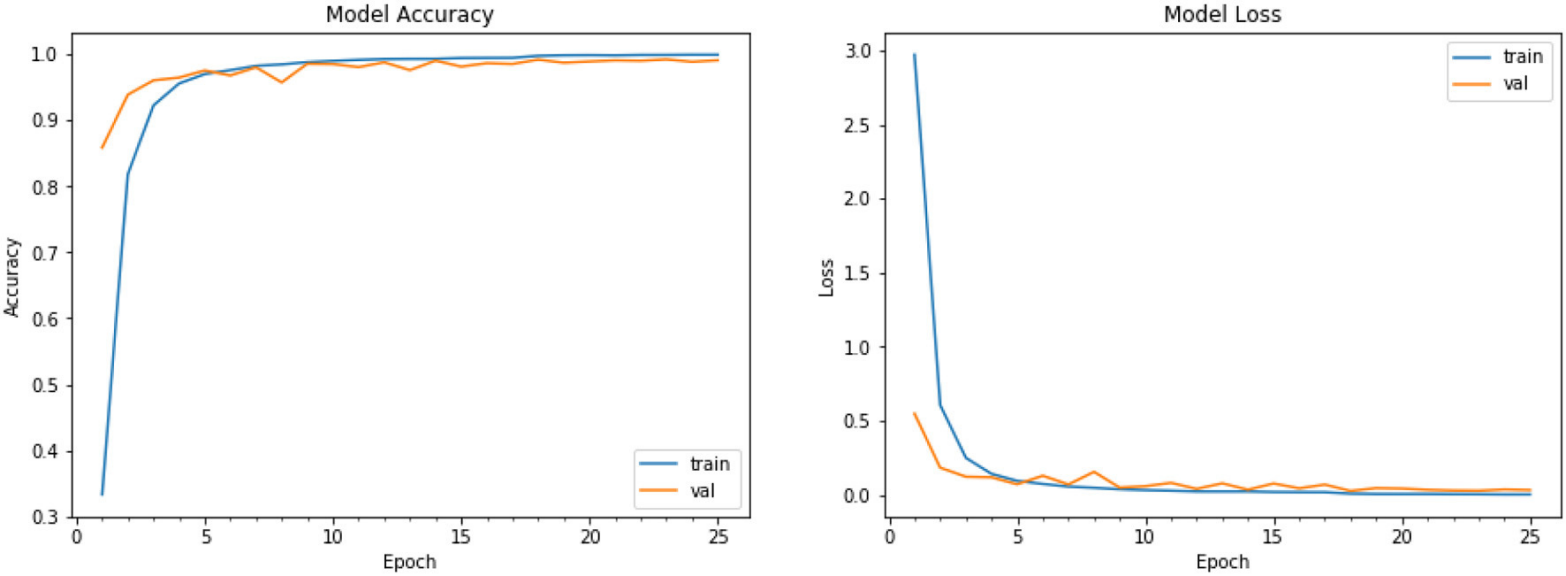
Training accuracy vs. validation accuracy (Left) training loss vs. validation loss (Right) performance graph.

**Figure 12:**
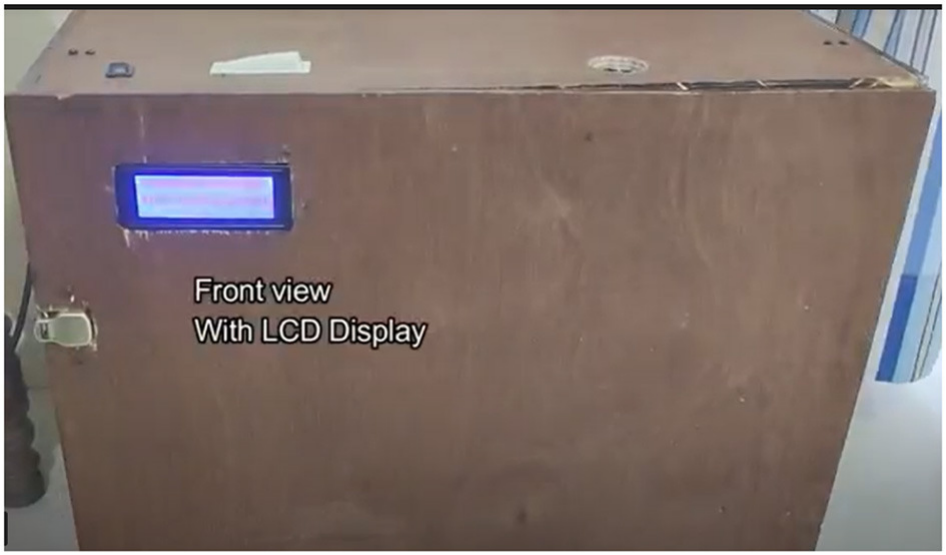
Device front view.

**Figure 13:**
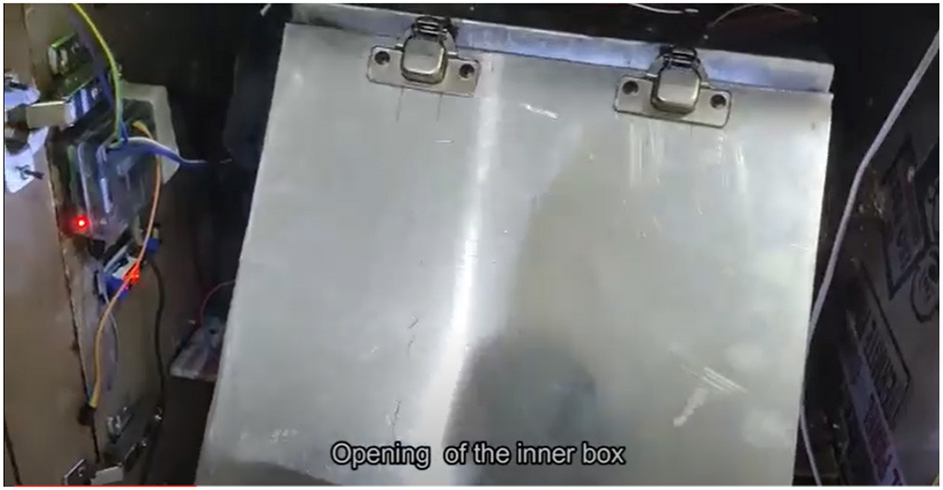
Device internal view.

**Figure 14:**
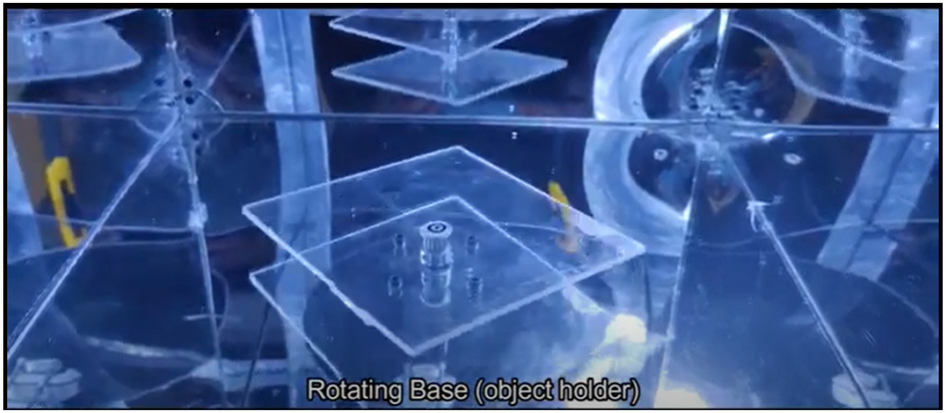
Device internal view - rotating base.

**Figure 15:**
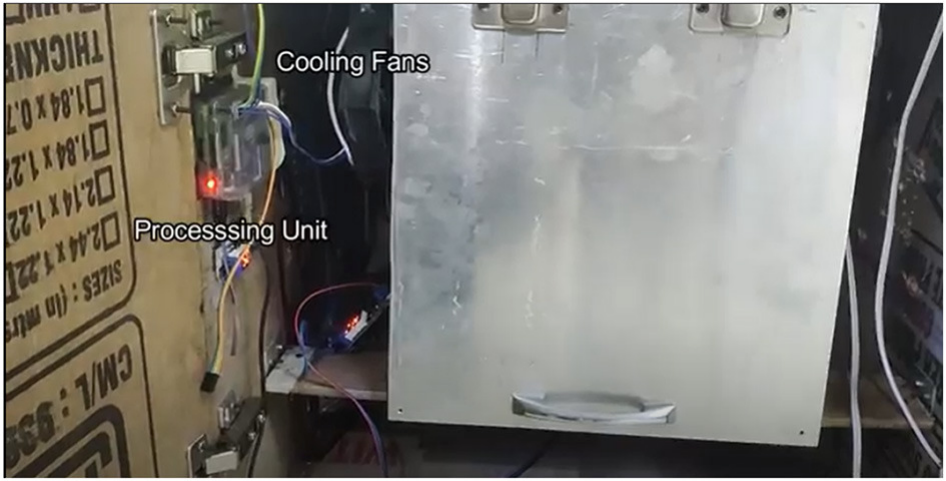
Device internal view - processing unit and cooling fans.

**Figure 16:**
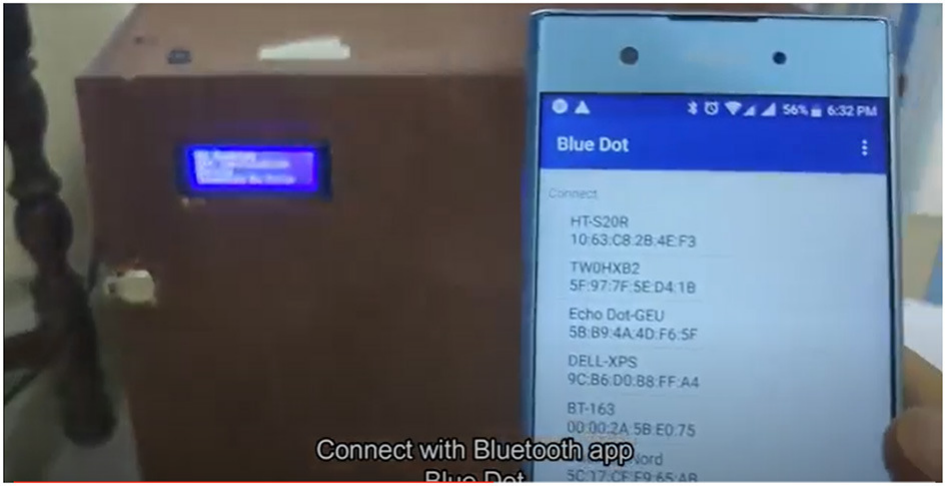
Device connectivity.

We have plotted the performance graph for our model for the training data and the validation data. We plot the graph between training data loss vs. validation data loss and training data accuracy and validation data accuracy (Figure 11) of the CNN model for object detection. The performance graph plot tells the increase in accuracy with the increase in the epochs and it also describes whether the model is trained properly or not. Figure 11 is the graph plotted between the loss at training and loss at Validation time. As portrayed in the graph, it is nearly converging so there is no overfitting and underfitting in our model. Training accuracy vs. validation accuracy performance graph shows that the model is trained nearly perfect as there is very little difference between the validation and the training accuracy. So, our model gives an accuracy of the 96.2% which is good enough to detect the type of vegetable and fruits from an image and it works fine for the single item or single fruit or vegetable image. It is also capable of predicting the class of multiple objects too. The model works fine for 131 types of categories. It gives very accurate results for the images input to the model. Finally, Figures 12–16 provide some additional images of the device under testing. These images are for illustration purposes only.

## 10. Conclusion and Future Scope

With the proposed system architecture, the edible items (especially raw fruits and vegetables) can be sanitized without losing their quality and without any damage to the cell body of the edible item. The AI-enabled device sanitizes the edible items with different optimal configurations made using object detection and automated configuration detection based on the detected class. The optimal configurations utilize the phenomenon of polarization filters which limits the expose of rays due to which the edible items get sanitized without losing their nutrition. Limited filter UVC doses are exposed uniformly throughout the surface of the item with different configurations with the optimal time.

In future, some points need to be considered to enhance the research work. The developed prototype is smart enough to detect the object inserted inside the box but the UVC 254 nm is not safe for humans as well as for edible items. So, 222 nm Germicidal UVC light can be used instead of 254 nm if it is available. Smart Home integration and API could be done to control the device using IoT, e.g., google assistant. Simultaneous sanitization could be performed for multiple items using optimal parameters.

## Data Availability Statement

Publicly available datasets were analyzed in this study. This data can be found here: https://www.kaggle.com/moltean/fruits.

## Author Contributions

UB and DK carried out the experiment. DK wrote the manuscript with support from UB. DK fabricated the device with the help from RA, AB, and MH. UB supervised the project. All authors contributed to the article and approved the submitted version.

## Conflict of Interest

The authors declare that the research was conducted in the absence of any commercial or financial relationships that could be construed as a potential conflict of interest.

## Publisher's Note

All claims expressed in this article are solely those of the authors and do not necessarily represent those of their affiliated organizations, or those of the publisher, the editors and the reviewers. Any product that may be evaluated in this article, or claim that may be made by its manufacturer, is not guaranteed or endorsed by the publisher.
